# Silver Nanoparticles Synthesized by Using the Endophytic Bacterium *Pantoea ananatis* are Promising Antimicrobial Agents against Multidrug Resistant Bacteria

**DOI:** 10.3390/molecules23123220

**Published:** 2018-12-06

**Authors:** Tahmina Monowar, Md. Sayedur Rahman, Subhash J. Bhore, Gunasunderi Raju, Kathiresan V. Sathasivam

**Affiliations:** 1 Unit of Microbiology, Faculty of Medicine, AIMST University, Kedah 08100, Malaysia; 2 Department of Biotechnology, Faculty of Applied Sciences, AIMST University, Kedah 08100, Malaysia; subhashbhore@gmail.com (S.J.B.); kathir.aimst@gmail.com (K.V.S.); 3 Regional Director, Ministry of Information, Government of the People’s Republic of Bangladesh, Gopalganj 8100, Bangladesh; sayed_radio@yahoo.com; 4 School of Distance Education, Universiti Sains Malaysia, Pulau Pinang 11800, Malaysia; rgunasunderi@usm.my

**Keywords:** endophytic bacterium, *Pantoea ananatis*, silver nanoparticles, pathogenic microbes, multidrug resistant bacteria

## Abstract

Antibiotic resistance is one of the most important global problems currently confronting the world. Different biomedical applications of silver nanoparticles (AgNPs) have indicated them to be promising antimicrobial agents. In the present study, extracellular extract of an endophytic bacterium, *Pantoea ananatis,* was used for synthesis of AgNPs. The synthesized AgNPs were characterized by UV–Vis spectroscopy, FTIR, transmission electron microscopy (TEM), scanning electron microscopy-energy dispersive X-ray spectroscopy (SEM-EDX), and Zeta potential. The antimicrobial potential of the AgNPs against pathogenic *Staphylococcus aureus* subsp. *aureus* (ATCC 11632), *Bacillus cereus* (ATCC 10876), *Escherichia coli* (ATCC 10536), *Pseudomonas aeruginosa* (ATCC 10145) and *Candida albicans* (ATCC 10231), and multidrug resistant (MDR) *Streptococcus pneumoniae* (ATCC 700677), *Enterococcus faecium* (ATCC 700221) *Staphylococcus aureus* (ATCC 33592) *Escherichia coli* (NCTC 13351) was investigated. The synthesized spherical-shaped AgNPs with a size range of 8.06 nm to 91.32 nm exhibited significant antimicrobial activity at 6 μg/disc concentration against *Bacillus cereus* (ATCC 10876) and *Candida albicans* (ATCC 10231) which were found to be resistant to conventional antibiotics. The synthesized AgNPs showed promising antibacterial efficiency at 10 µg/disc concentration against the MDR strains. The present study suggests that AgNPs synthesized by using the endophytic bacterium *P. ananatis* are promising antimicrobial agent.

## 1. Introduction

Antibiotic resistance is a great threat to global health. Although it occurs naturally, irrational and indiscriminate uses of antibiotics are accelerating the process resulting in a grave dilemma in treating a number of infections due to fact the antibiotics that are conventionally used to treat them are becoming less effective [1,2,3]. Since the 1950s, both the legitimate and irrational uses of antibiotics have led to increase in antibiotic-resistant bacteria. Until recently, a series of epidemics have been noted marked in the antibiotic era caused by several resistant organisms such as penicillin-resistant *Staphylococcus aureus*, methicillin-resistant *Staphylococcus aureus* (MRSA), vancomycin-intermediate *Staphylococcus aureus* (VISA), drug-resistant *Vibrio cholerae*, multidrug-resistant (MDR) and extensively drug-resistant (XDR) *Mycobacterium tuberculosis*, CTX-M *β*-lactamases resistant *Escherichia coli* and *Klebsiella pneumoniae*, and many others [4,5,6,7,8,9]. Drug-resistant pathogens are now common in hospitals and other health care settings as well as in communities and the environment [2].

It is estimated that by the 2050, the production of antimicrobials will be one million metric tons in comparison to nearly 25 million pounds manufactured in 2000 in the USA alone. Russia, China, and India are producers of the largest quantities of these compounds [10]. Antimicrobial resistance (AMR) genes are common in pristine natural environments, and genotypic or phenotypic resistance has been found in many environmental bacterial isolates. AMR is acquired by horizontal gene transfer or by mutation [11]. AMR can also be developed via an evolutionary process producing a microbial community with the resistance genes and spread of mobile genetic elements to produce microbial resistance to susceptible genes [12]. Furthermore, some trigger points like overuse, misuse, travel, migration, socioeconomic factors and environmental antibiotic pollution all contribute the ineffectiveness of the drugs in the populations affected by the global challenges of AMR [2,12,13,14,15].

Nowadays, the emerging AMR is an alarming global public health threats estimated to be responsible for over 700,000 deaths worldwide [13]. In USA, about 2 million people are infected with drug resistant pathogens and of these, about 23,000 die per year, whereas in Europe, the number of deaths due to multidrug resistant bacteria (MDR) is about 25,000 per year [15]. The incidence of the AMR has been considered as an alarming concern in Europe (59%), followed by South-East Asia (18%), Western Pacific (12%), Americas (6%), and Africa (6%) [16]. Due to impending threats of MDR bacterial infections, a total of deaths per year were 23,000 in the USA, 25,000 in EU repetitively and 58,000 in India [17]. There are about 440,000 emerging MDR-TB cases per year, among them approximately 150,000 patients die each year all over the world [18] and it is estimated that about 10 million people may die every year by 2050 due to multidrug resistant (MDR) infections [15]. In the USA, nosocomial infections by the drug resistant pathogens cost thousands of lives and 125 billion $US extra hospital charges per year [19]. If the present trend continues without suitable proper strategies then it will have a tremendous impact on human and economic cost by 2050 leading to 10 million deaths every year repetitively with an approximate reduction of 2% to 3.5% in Gross Domestic Product (GDP). The consequences of these drug resistant infections would cost the world up to 100 trillion USD [20]. Research and development of new antibiotics are among the major interventions with a view to prevent and control the spread of AMR [3], however, several studies have suggested that AgNPs can be used in combating infections caused by MDR bacterial strains [21,22,23,24].

In the recent times, applications of silver nanoparticles (AgNPs) have gained attention for their important role in nanoscience and nanotechnology, especially in nanomedicine. AgNPs are now being used in a broad spectrum of scientific fields including biotechnology, biomedicine, pharmacy, ecology, and electronics with further possible uses in agriculture, veterinary medicine, food, cosmetics, textiles, construction and other industries. Modern approaches of using AgNPs are promising in terms of exploring and formulating a diverse range of new drugs with antibacterial and antifungal activities [25,26]. Furthermore, AgNPs have been suggested to be materials of the future. The current global market for nanomaterials has been estimated to range from 0.3 million tons up to 1.6 million tons among which the Asian region which accounts for approx. 34% has the largest market share, followed by North America (approx. 31%) and Europe (approx. 30%), respectively [27].

As opposed to the beneficial role of the AgNPs, one grave concern about the use of these nanoparticles is their inherent toxicity. The release of AgNPs into the aquatic environment in the form of Ag^+^ through dissolution process can affect human health and the environment. The adsorption properties of natural organic matter affects their dissolution properties reducing the release of Ag^+^ in a dose-dependent manner and thus modulating or even inactivating the toxicity of AgNPs [28]. Furthermore, AgNPs can produce many reactive oxygen species (ROS), including superoxide radical (O_2_^−^), hydroxyl radical (OH^−^), hydrogen peroxide (H_2_O_2_), and singlet molecular oxygen (^1^O_2_), which in turn can induce DNA and protein damage and lipid peroxidation. These facilitate the antimicrobial effects at the same time they cause significant cytotoxicity in mammalian cells [28,29]. On the other hand, AgNPs functionalized with different molecules such as DNA/RNA may selectively target different cells and antibodies or polymers can be used to extend the half-life time for in vivo circulation in drug and gene delivery applications [28].

Different physical, chemical and biological methods are applied to synthesize AgNPs [25,26,27,29]. The chemical and physical methods are expensive and incompatible with a sustainable ecosystem due to their limitations such as the requirement for large amounts of energy and the generation of toxic by-products. On the contrary, biological methods, also termed green chemistry approaches, have been suggested for the facile synthesis of AgNPs. Synthesis of AgNPs following biological methods are simple, rapid, cost effective, non-toxic, and environmentally friendly. Besides, AgNPs with well-defined size and shape can be produced under optimized conditions. High density, stability, and the ready solubility of prepared AgNPs in water are some other advantages of the biological methods [30]. A variety of biomaterials including bacteria, fungi, plants, and small biomolecules like vitamins and amino acids have been reported to be used for the synthesis of AgNPs [25,26,30,31]. A recent study demonstrated that the microbial inhibitory and cellular toxicity of green-synthesized silver nanoparticles depends on the applied natural extracts. It has been reported that AgNPs synthesized by using coffee extract show substantial antimicrobial inhibitory action while being non-toxic to human and mouse cells [32]. This study opens up a new dimension in the field of nanotechnology suggesting the fact that green synthesis of AgNPs using various biological materials can be a promising and safer alternative source with defined biological activity.

Synthesis of AgNPs in solution generally requires a metal precursor, reducing agents, and a stabilizing/capping agent. In the biological methods, biomolecules obtained from living organisms such as plants, bacteria, fungi, yeast, and algae act as the reducing and capping agents [33]. Numerous biomolecules such as phenolics, flavonoids, terpenoids, polysaccharides, alkaloids, proteins, enzymes, amino acids, carbohydrates, vitamins, alcoholic compounds, phytosterols, quinol and chlorophyll pigments, linalool, methyl chavicol, eugenol, caffeine, theophylline, ascorbic acid, carboxylic acids, flavonols, flavonones, saponins, tannins, triterpenes, sugars, sulfhydryls, quinones, etc. affect the reduction and the capping of the nanoparticles [30,34]. These biomolecules have unique chemical properties in reducing and effectively wrapping nanoparticles that help to preventing their agglomeration. Hydroxyl and carboxyl groups of phenolic compounds are able to bind to metals. The polyphenols act both as the reducing and capping agents. The capping layer of polyphenols reduce Ag^+2^ to Ag^+0^ and the oxidized polyphenol binds to the AgNPs via −C=O bonds and also simultaneously stabilizes them [34]. However, some recent literature suggest that sunlight-mediated green synthesis of AgNPs is the most facile way [22,35,36,37].

Endophytes are microorganisms inhabiting in the tissues of living plants provide a large variety of bioactive secondary metabolites which have potential use in the medical, agricultural and industrial sectors [38,39,40]. Exploration of the multidimensional uses of endophytic bacteria is highly important across the world with a view to deal with the increasing number of drug resistant bacteria [41]. Endophytic bacteria are a less explored biomaterial that can be used to synthesize AgNPs. Bacterial proteins or amino acids act as reducing agents in the AgNPs synthesis process, and the shape, size and monodisperity of the AgNPs can be controlled [26]. The major advantage of bacteria-based AgNPs synthesis is their large scale sustainable production with minimal use of toxic chemicals [42].

The current prospects for replacing current antimicrobial drugs are not healthy, and a global strategy is required to enact the 10×^′^20 initiative and thus create a stable research infrastructure for antimicrobial drug development [17]. In this context, the present work was therefore aimed to synthesize AgNPs using an endophytic bacterium, *P. ananatis*, and to evaluate their antimicrobial efficiencies against two Gram-positive and two Gram-negative bacteria, one fungus and four MDR bacteria. To the best of our knowledge, extracellular synthesis of AgNPs using this endophytic bacterium, and the antimicrobial potentials of the synthesized AgNPs against a range of pathogenic and MDR microbes are reported here for the first time.

## 2. Results and Discussion

### 2.1. UV-Vis Spectrometry

**Figure 1:**
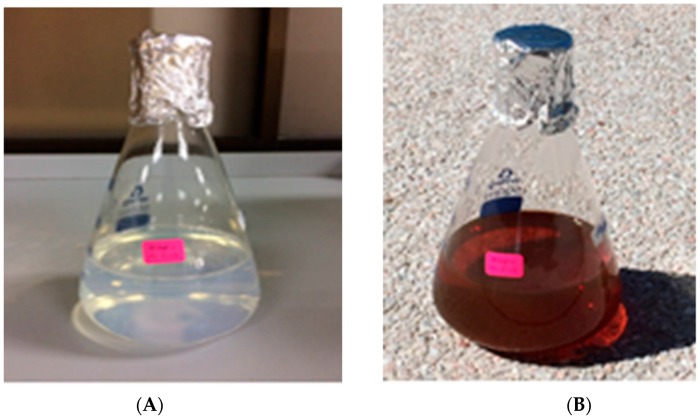
Visible color change of *P. ananatis* CFE with AgNO_3_ before (**A**) and after (**B**) synthesis of AgNPs.

**Figure 2:**
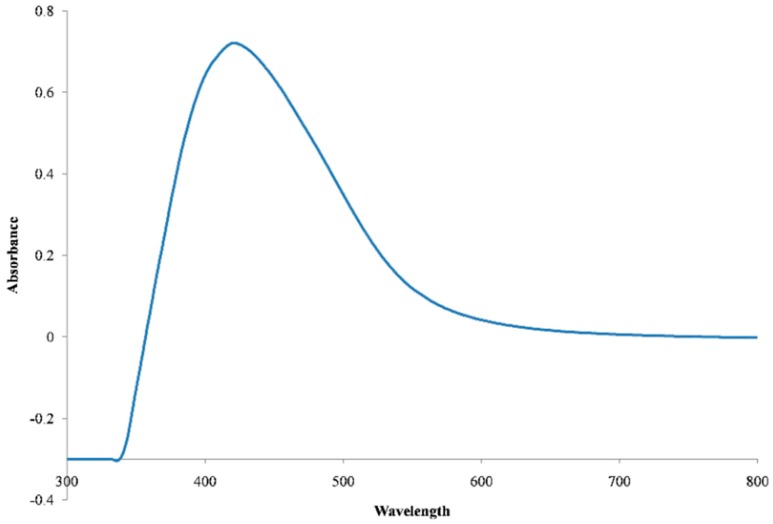
UV-Vis spectra of the synthesized AgNPs.

Formation of AgNPs in the reaction mixture exposed to sunlight produced a gradual color change (Figure 1) due to the excitation of strong surface plasmon vibration with the AgNPs in the visible region [43,44,45]. The synthesized AgNPs exhibited a clear surface plasmon resonance resulting in the formation of a sharp peak at 421 nm wavelength in the UV-visible spectra (Figure 2), which was specific for the synthesized spherical AgNPs [46,47]. The peak intensity was found to increase with the increase of exposure time under the experimental conditions due to the increased synthesis of the AgNPs in the reaction mixture [21,46]. On the other hand, the reaction mixture kept in the dark exhibited only a slight change in color, which clearly indicated that the photocatalytic action of the sunlight played an important role in the AgNPs synthesis process. The control was found to exhibit no change in color under exposure to sunlight, which provides insight into the fact that the formation of AgNPs requires both exposures to sunlight and cell free extract (CFE). Therefore, it is evident from the present study that *P. ananatis* is able to synthesize AgNPs, rapidly and easily.

### 2.2. FTIR Study

**Figure 3:**
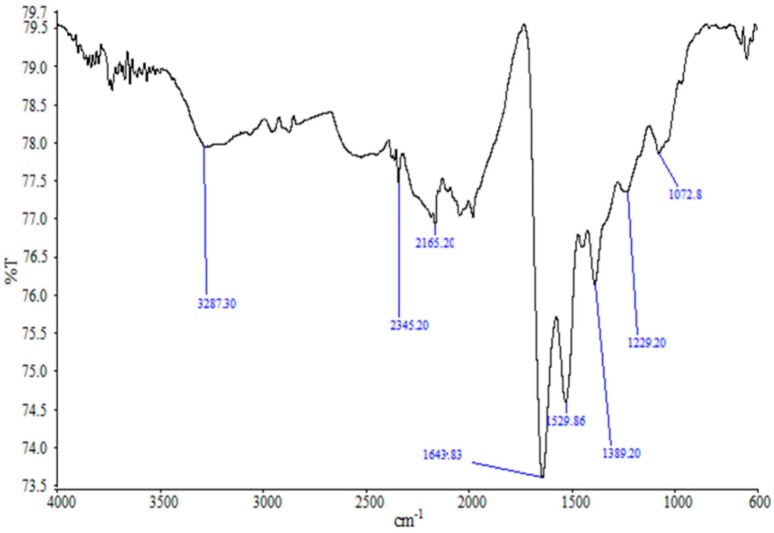
FTIR spectra of the synthesized AgNPs.

The FTIR spectra of the synthesized AgNPs (Figure 3) showed absorption peak at 3287.30 cm^−1^, which represents N−H functional groups of protein and OH stretching vibrations of phenols/alcohols. The band at 1643.83 cm^−1^ is due to C−O stretching in proteins. The peak at 1389.20 cm^−1^ is attributed to the symmetric deformation of the CH_3_ vibration, and the bands at 1229.20 cm^−1^ and 1072.8 cm^−1^ can be assigned to the C−O stretching vibrations. The peaks at 2345.20 cm^−1^ and 2165.20 cm^−1^ correspond to the asymmetric C−H stretching vibration of aliphatic groups, and C=O stretching vibrations of carboxylic acids, respectively. These findings reveal the interaction of the functional amino groups with the surface of AgNPs, which might have acted as the capping area for the stability of the synthesized AgNPs [21,43,44,45,48]. The FTIR peaks also reveal the presence of phenols, proteins and carboxylic acids in the CFE, which might have acted as the reducing and stabilizing agents in the synthesis of AgNPs [30,34].

The endophyte ecological niche is a hot spot for horizontal gene transfer (HGT). The HGT among plant-associated endophytic bacteria was first reported by Taghavi and co-workers [49]. It is assumed that bacteria capture genetic material from plants [50]. Subsequently, several authors have demonstrated and described the HGT to endophytes from their host plants [51,52,53,54,55]. Pathways for and the mechanism of HGT have been described in details in the literature [56]. Endophytes are well known for producing diverse range of secondary metabolites [57]. It has earlier been reported that endophytic microbes have the ability to produce the same or similar pharmacologically active secondary metabolites as their hosts [58]. The presence of the biomolecules found in the FTIR study might be due to the HGT of the responsible genes from the host plants.

### 2.3. TEM Study

**Figure 4:**
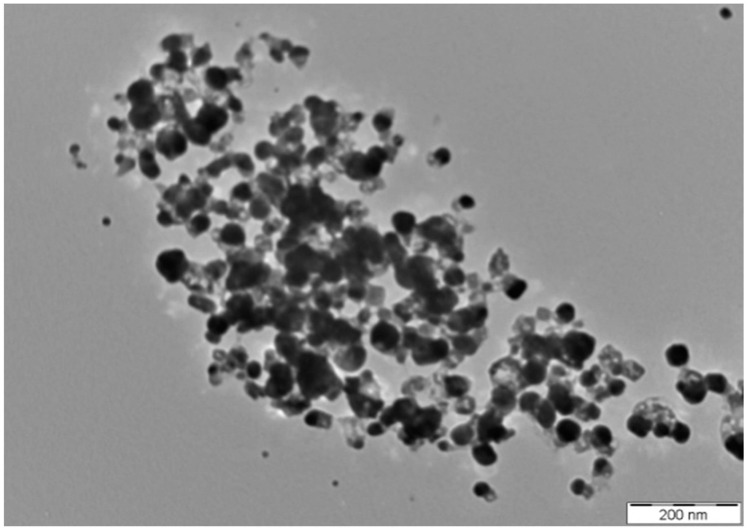
TEM image of the synthesized AgNPs.

The size and shape of synthesized AgNPs greatly affect their antimicrobial activity [59]. The TEM image analysed for determining the size and shape of the synthesized AgNPs is presented in Figure 4. The size of the synthesized AgNPs was found to fall in the range of 8.06 to 91.32 nm with a mean size of 35.02 ±13.41 nm. The synthesized AgNPs were mainly spherical in shape, and were identical to other AgNPs previously reported in literature [43,44,60,61,62]. The AgNPs synthesized in the present study were inconsistent in size and polydispersed [45,63,64,65].

### 2.4. SEM-EDX Studies

**Figure 5:**
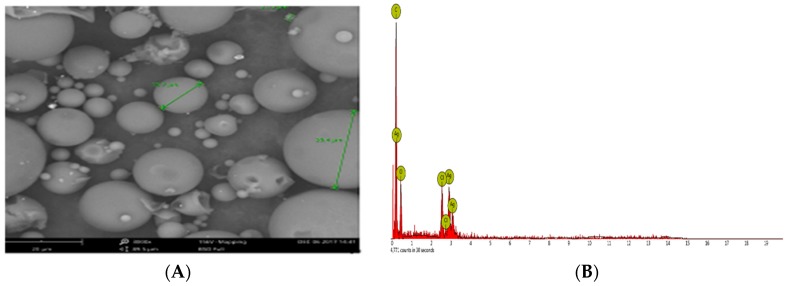
(**A**) SEM image, (**B**) EDX image of the synthesized AgNPs.

The SEM image of the synthesized AgNPs (Figure 5A) further confirmed the spherical shape and size of the nanoparticles ranging from 8.06 to 91.32 nm. The quantitative elemental composition of the synthesized AgNPs (Table 1) was indicative of the presence of silver without impurities. As shown in the Figure 5B, the elemental ratio of Ag was observed in different parts of synthesized AgNPs in the EDX spectrum [64]. A strong Ag atom signal was observed in the synthesized AgNPs in comparison to other signals from carbon, oxygen and chlorine (Figure 5B). Carbon and oxygen signals were obtained from the protein biomolecules existing in the sample due to X-ray emissions [66]. The other elements except silver detected in the EDX might have been derived from the grid and detectors [64]. Like the present study, a number of previous studies have reported the existence of silver including elemental composition without any impurity after performing SEM-EDX analyses [60,65,67]. In the present study, the EDX spectrum confirmed the synthesis of AgNPs using *P. ananatis*, is in good agreement with the previous studies reported in literature [45,60,67,68].

### 2.5. Zeta Potential Study

**Figure 6:**
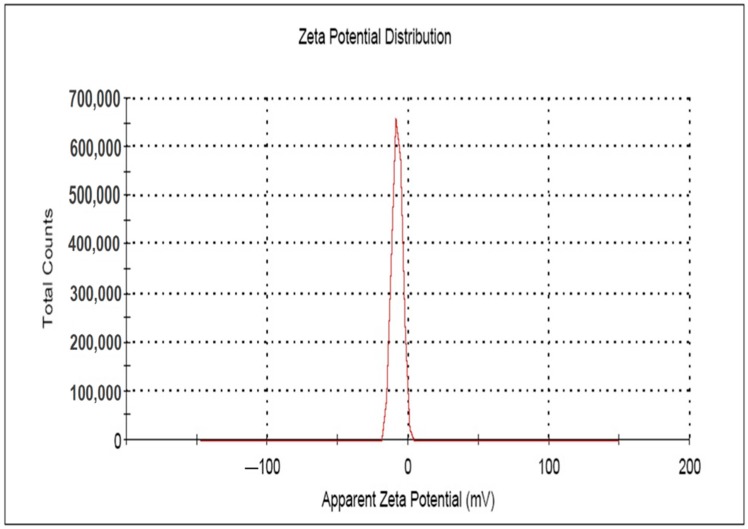
Zeta potential image of the synthesized AgNPs.

The surface charge potential, described as the Zeta potential, plays a significant role in terms of the stability of AgNPs in aqueous solution. The long-term stability of colloidal AgNPs is monitored spectroscopically by the Zeta potential technique [69], which indicates the changes in surface charge with time. Such a method is widely used to control the stability of colloidal metal nanoparticles. The stability towards agglomeration will be higher as the zeta value increases [44,46,48]. The Zeta potential value of AgNPs is found higher in alkaline pH due to the electrostatic repulsion of the particles resulting from adsorption of OH^−^ ions on the AgNPs, whereas at acidic pH, low or negative values are observed due to the absence of OH^−^ ions [46]. In the present study, the Zeta potential values of the synthesized AgNPs (Table 2, Figure 6) observed in triplicate were found to be negative, due to the presence of a high number of negatively charged functional groups on the surface of the synthesized AgNPs [44]. The mean of the zeta potential value was found as −7.48 ± 0.64 mV, which strongly supports the formation of moderately stable colloidal AgNPs [44,45,48,65,70].

### 2.6. Antimicrobial Assay

**Figure 7:**
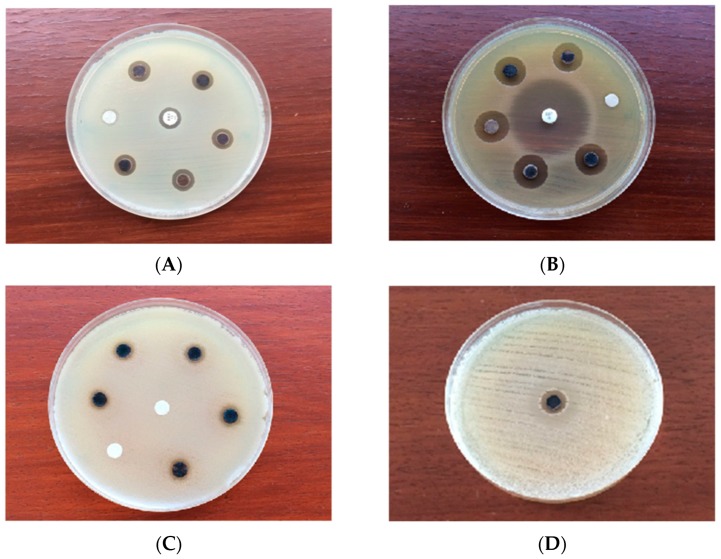
Antimicrobial activity of the synthesized AgNPs against (**A**) *S. aureus* subsp. *aureus* (ATCC 11632) (**B**) *E. coli* (ATCC 10536) (**C**) *C. albicans* (ATCC 10231) (**D**) MDR *E. faecium* (ATCC 700221).

The synthesized AgNPs exhibited promising antimicrobial efficiency against pathogenic microbes and MDR bacteria (Table 3, Figure 7A–D) in a dose-dependent manner for different microorganisms [45]. The zones of inhibition (ZOI) displayed by the synthesized AgNPs for different pathogenic microorganisms in the present study was significantly different (*p* < 0.05). The synthesized AgNPs exhibited notable antibacterial activity against the Gram positive bacteria *S. aureus* subsp. *aureus* (ATCC 11632) and *B. cereus* (ATCC 10876) with ZOIs of 11.30 ± 0.07 mm (MIC: 2.75 μg/mL) and 9.16 ± 0.05 mm (MIC: 2.25 μg/mL), respectively. *S. aureus* subsp. *aureus* (ATCC 11632) was less effective (ZOI: 10.14 ± 0.05 mm) while *B. cereus* (ATCC 10876) was resistant to the conventional antibiotic, ampicillin (10 μg/disc). On the other hand, the synthesized AgNPs exhibited lower ZOIs of 15.12 ± 0.08 mm (MIC: 3.25 μg/mL) and 8.02 ± 0.08 mm (MIC: 1.75 µg/mL) against the Gram negative bacteria *E. coli* (ATCC 10536) and *P. aeruginosa* (ATCC 10145), respectively, in comparison to ciprofloxacin (5 μg/disc) in the positive control group. The synthesized AgNPs were found to exhibit a pronounced antibacterial activity against MDR strains of *S. pneumonia* (ATCC 700677), *E. faecium* (ATCC 700221), *S. aureus* subsp. *aureus* (ATCC 33592) and *E. coli* (NCTC 13351) with ZOIs of 10.20 ± 0.07 mm (MIC: 2.75 μg/mL), 12.16 ± 0.05 mm (MIC: 2.25 µg/mL) and 12.24 ± 0.05 mm (MIC: 3.5 µg/mL), respectively. No significant difference was found (*p* < 0.05) in the mean ZOI with the synthesized AgNPs between *E. faecium* (ATCC 700221) and *E. coli* (NCTC 13351), and *S. pneumoniae* (ATCC700677) and *S. aureus* subsp. *aureus* (ATCC33592). On the other hand, the antifungal activity of the synthesized AgNPs against the pathogenic fungus, *C. albicans* (ATCC 10231) was promising with a ZOI of 7.16 ± 0.09 mm (MIC: 1.75 µg/mL), while the pathogen was found to be resistant in the control group treated with itraconazole (10 µg/disc). It was evident from the study that although *B. cereus* (ATCC 10876) and *C. albicans* (ATCC 10231) were found to be resistant against the conventional antibiotics, the synthesized AgNPs were found to be effective against those pathogenic microbes, resulting in promising ZOIs at the experimental level of concentrations. Neither the extracellular extract not silver nitrate solution was found to exhibit any antimicrobial efficiency against the tested microbes in the present study under the experimental conditions. This can be attributed to the absence of any active compound possessing antimicrobial activity in the extracellular extract and silver nitrate at the experimental concentration level was not able to produce any antimicrobial effect against the tested microorganisms [71]. Therefore, it is suggested that the AgNPs synthesized by the endophytic bacterium, *P. ananatis* in the present study can be more promising antimicrobial agents against a range of pathogenic and MDR microbes, including *C. albicans* (ATCC 10231) and *E. coli* (ATCC 10536) than the conventional antibiotics.

Green synthesized nanoparticles are generally more toxic than those obtained using physical and chemical methods. The toxicity of nanoparticles is reported at cellular, subcellular and biomolecular levels. The in vitro and in vivo cytotoxicity of AgNPs and the mechanisms involved have been reviewed by several authors [30,72]. Agglomeration and recrystallization of AgNPs have been reported to reduce their toxicity [62]. No induced genetic toxicity in male and female rat bone marrow was observed for exposure to AgNPs at a high dose of 2.9 × 10^6^ particles/cm^3^ for 6 h/day by inhalation for 90 days in vivo [73]. Earlier it has been reported that AgNPs of different size and shape can impart equal cytotoxicity while exhibiting different antibacterial effects due to the differences in the dissolution rate. Hence, the exploration of the dissolution rate difference of AgNPs has been suggested for application of these nanoparticles with a relatively higher bacterial effect while minimising cytotoxic effects towards normal tissues [74].

It has been reported earlier that AgNPs have potential antimicrobial efficiency against MDR microbes [66] as well as pathogenic microbes [21,44,45,75,76]. It is therefore, understood that the AgNPs synthesized in the present study could lead to a more significant antimicrobial efficiency against a wide range of pathogenic microbes and MDR pathogens at a higher level of concentration. In that instance, it is advised to consider the in vivo toxicity of the AgNPs as they may cause genotoxic and epigenetic changes in human beings [77] and impairment to ecosystems [78]. However, to the best of our knowledge, the present study is the first ever to report on the potential of AgNPs against these pathogenic microbes and MDR bacteria.

There are several factors that affect the antimicrobial activity of AgNPs. Size [30,44], shape and surface charge of AgNPs, species sensitivity and tolerance to AgNPs [44], bacterial type, genus and species [45], concentration, pH of the medium and time of exposure to the pathogens [30] all have a significant role in the exhibited antimicrobial activity of AgNPs. Cells wall of Gram-positive bacteria possess a thick peptidoglycan layer composed of linear polysaccharide chains cross-linked by short peptides that results in a rigid structure that hinders the penetration of AgNPs into the bacterial cell wall as compared to Gram negative bacteria where the cell wall consists of a thinner peptidoglycan layer [45,66,76]. Two possible mechanisms of action have been described through which AgNPs exhibit antimicrobial effects against bacterial cells: (a) membrane damage through association/interaction of AgNPs with DNA and biomolecules leading to inhibition of cell multiplication, and (b) ROS formation through interaction with enzymes and or biomolecules leading to cell damage/destruction [30]. However, from the present study, it could be hypothesized that the exposure of the microorganisms, including fungi, to different AgNPs concentrations might have damaged the DNA backbone of the microorganisms resulting in microbial cell death [21].

The results of the present study are more promising than the works of Mathew and co-workers who synthesized AgNPs using *Zingiber officinale* rhizome extract in the presence of sunlight and reported the resulting AgNPs to exhibit excellent antibacterial activity against *Staphylococcus aureus* and *Escherichia coli* with MIC values of 62.5 μg/mL and 125 μg/mL, respectively [22]. In an earlier study, it has been reported that the AgNPs synthesized by a novel actinobacterium, *Sinomonas mesophila* MPKL 26, shows good antibacterial activity against MDR strains of *Staphylococcus aureus* with a ZOI of 12 mm using the well diffusion method at a working concentration of 1560 µg/mL [36]. That concentration was three fold higher than in the present study (500 µg/mL), and with respect to the ZOI, the AgNPs synthesized in the present study using the endophytic bacterium, *P. ananatis* are therefore suggested to be a better option in dealing with MDR strains.

## 3. Materials and Methods

### 3.1. Chemicals, Endophytic Bacteria and Microbial Strains

Analytical grade AgNO_3_ solution and KBr powder (Fisher Scientific, Hampton, NH, USA), ampicillin (10 µg/disc) and ciprofloxacin (5 µg/disc) antibiotic discs (BD BBL Sensi-Disc, Beckton Dickinson, Franklin Lakes, NJ, USA); antifungal itraconazole powder (Tokyo Chemical Industry, Tokyo, Japan); Luria Bertani Broth, nutrient broth, nutrient agar, potato dextrose broth, potato dextrose agar and Mueller-Hinton agar (HiMedia Lab. Ltd., Mumbai, India) were used in the present study. The endophytic bacteria, *Pantoea ananatis* (GenBank accession no. HQ650772), identified earlier by 16S rRNA sequencing [79], was obtained from the laboratory of the Faculty of Applied Sciences, AIMST University, Malaysia. Pathogenic bacterial strains of *Staphylococcus aureus* subsp. *aureus* (ATCC 11632), *Bacillus cereus* (ATCC 10876), *Escherichia coli* (ATCC 10536), *Pseudomonas aeruginosa* (ATCC 10145), fungal strains of *Candida albicans* (ATCC 10231) and MDR strains of *Streptococcus pneumoniae* (ATCC 700677), *Enterococcus faecium* (ATCC 700221), *Staphylococcus aureus* subsp. *aureus* (ATCC 33592), *Escherichia coli* (NCTC13351) were obtained from Bio-Focus Saintifik Sdn Bhd, (Selangor, Malaysia).

### 3.2. Culture of the Endophytic Bacteria and Preparation of Cell Free Extract

Culture of the endophytic bacteria maintained on a Luria Bertani Broth (LBB) as glycerol stocks at −80 °C was used in the present study. Cell free extract was prepared following the methods described in literature [80] with some modifications. For inoculum preparation, a loopful of the culture grown on nutrient broth (37 °C for 24 h) was inoculated into 250 mL sterile Luria Bertani broth and incubated at 37 °C for 24 h in a rotary incubator shaker (Innova 40, New Brunswick Scientific, New York, CO, USA). After 24 h, the culture was taken into a 50 mL Beckman tube (Beckman Coulter, Inc., Pasadena, CA, USA) and centrifuged using a high speed centrifugal machine (AvantiJ-26 XPI, Beckman Coulter, Inc.) at 8000 rpm for 10 min at 4 °C. After centrifugation, cell-free extract (CFE) was collected in a sterile beaker and used immediately for AgNPs synthesis.

### 3.3. Synthesis of AgNPs

Synthesis of AgNPs was performed following the method outlined in literature [36] with some modifications. In brief, the reaction mixture of freshly prepared cell free extract and 100 mL of 0.1 mM AgNO_3_ solution (2%, *v/v*) was exposed to bright sunlight. The pH of the reaction mixture was adjusted to neutral. The temperature of the ambient environment and solar intensity were 32 °C and ~72,000 lux, respectively. The solar intensity was measured using a mobile app (Light Meter^©^ Vlad Polyanskiy Luxmeter (Voronezh, Pirogova 37, Russia) installed in an iOS device. Formation of AgNPs was monitored at regular time intervals through investigating the pattern of surface plasmon resonance band using a UV-Visible spectrophotometer (DU-800, Beckman Coulter, Inc.). Simultaneously, similar experiment was conducted under dark conditions (25 °C, 0 lux) for 24 h. AgNO_3_ solution (0.1 mM) without CFE was used as the control. The synthesized AgNPs in the reaction mixture were centrifuged at 12,000 rpm for 15 min at 4 °C in the high speed centrifugal machine. Repeated washing (×3) was done with deionized water to eliminate the water soluble biological molecules, other metabolites and impurities. The final mass of the AgNPs was collected after vacuum drying (Yamato Scientific Co. Ltd., Tokyo, Japan).

### 3.4. Characterization of AgNPs

Characterization of the synthesized AgNPs was carried out using UV-Vis spectrometry [36,44,80], FTIR spectrometry [36,44], TEM [36,80], SEM-EDX [80], and Zeta potential [44]. The formation of AgNPs was confirmed by the UV-Vis spectrum of the reaction mixture. For this, the reaction mixture was taken into a 1 cm path quartz cell, and scanned at a resolution of 1 nm from 200 to 800 nm using the UV-Vis spectrophotometer.

The FTIR analysis was carried out to find out possible functional biomolecules responsible for the reduction of Ag^+^ to AgNPs. For this, the dried AgNPs was ground with KBr pellets, and scanned the spectrum ranging from 400–4000 cm^−1^ at a resolution of 4 cm^−1^ using a FTIR spectrophotometer (PE 1600, GMI Inc., Ramsey, MN, USA).

Morphology, size and distribution of the synthesized AgNPs was observed using TEM. Prior to analysis, AgNPs were diluted with deionized water and sonicated in a water bath for 5 min. A drop of the diluted AgNPs solution was placed on a carbon coated copper grid. The sample was kept under infrared light to dry before loading them onto a specimen holder. TEM images were taken by analysing the prepared grid on a Philips CM 12 TEM system (Philips Electron Optics, Eindhoven, The Netherlands) equipped with the Philips Docu Version 3.2 image analysis software (Philips Electron Optics, Eindhoven, The Netherlands) at 120 kV.

Further morphological analysis of the synthesized AgNPs was performed using SEM-EDX (Phenom-World B.V., Eindhoven, The Netherlands). For this, a drop of the freshly synthesized AgNPs was dried on a glass slide for analysis under SEM, and the presence of elemental silver was confirmed through EDX.

Zeta potential was carried out to measure the magnitude of the electrostatic or charge repulsion or attraction among the nanoparticles and to determine the stability of the AgNPs using Zetasizer, ver. 7.11 (Malvern Instruments Ltd., Malvern, UK).

### 3.5. Antimicrobial Assay

The antibacterial activities of the synthesized AgNPs against the pathogenic microbial strains of *S. aureus* subsp. *aureus* (ATCC 11632), *B. cereus* (ATCC 10876), *E. coli* (ATCC 10536), *P. aeruginosa* (ATCC 10145), and MDR strains of *E. coli* (ATCC 25922), *S. aureus* (ATCC 25923) and *P. aeroginosa* (ATCC 27853) were performed using the disk-diffusion method [81] following the CLSI guidelines [82]. An ampicillin disc (10 μg) for the Gram positive bacteria, *S. aureus* (ATCC11632) and *B. cereus* (ATCC10876), and a ciprofloxacin disc (5 μg) for the Gram negative bacteria, *E. coli* (ATCC10536), *P. aeruginosa* (ATCC10145) were used as positive controls. The antifungal activity of the synthesized AgNPs against *C. albicans* (ATCC10231) was tested using the disk-diffusion method [81] following the CLSI guidelines [83], where itraconazole (10 µg) was used as the positive control.

The pathogenic and MDR bacteria were grown on nutrient broth (24 h at 37 °C) while fungal isolates were grown on potato dextrose broth (24 h at 30 °C) in sterile screw-cap test tubes. Thereafter, the pathogenic and MDR microbes were cultured onto nutrient agar (24 h at 37 °C) while fungal isolates were cultured onto potato dextrose agar (24 h at 30 °C) in Petriplates. Each of the microorganisms maintained at 0.5 McFarland turbidity was swabbed on a Petriplate containing Mueller-Hinton agar (MHA) for the antimicrobial susceptibility test following the disk-diffusion method [81]. AgNPs concentrations of 300 µg/mL (6 µg/disc) and 500 µg/mL (10 µg/disc) were impregnated on sterile 6 mm diameter of Whatman No. 1 filter paper (Sigma-Aldrich, St. Louis, MO, USA) discs for the pathogenic microbes and MDR bacteria, respectively. The antibiotic discs were placed in the MHA and incubated for 24 h at 37 °Cfor bacterial isolates and at 30 °Cfor fungal isolates. The zone of inhibition (ZOI) was measured in mm with a Vernier calliper (Mitutoyo, S-530, Mitutoyo Co., Kawasaki, Japan). Simultaneously, similar experiments were carried out using the freshly prepared extra cellular extract and silver nitrate solution as the negative control. All the experiment was done in triplicate, and the results were expressed as mean ± SD.

The MIC was estimated by the broth dilution method [82]. A range of AgNPs concentrations was tested against the microorganisms for standardization [84]. Different concentrations of the synthesized AgNPs were made with 4 mL NB following two-fold serial dilution. A 0.2 mL aliquot of McFarland standard suspension of bacteria was added in each test tube. The positive control and negative control were also prepared in the sterile test tube with 2 mL NB mixed with 0.2 mL McFarland standard suspension of bacteria and NB, respectively. Then all the test tubes were incubated at 37 °C for 24 h for the pathogenic bacteria, and at 30 °C for 24 h for the *Candida albicans*. The lowest dilution of the synthesized AgNPs from the endophytic bacteria showing the absence of growth by exhibiting no turbidity was determined as the MIC of the respective endophytic bacteria. The experiments were tested in triplicate.

### 3.6. Statistical Analysis

All the experiments were performed in triplicate and the results were expressed as mean ± SD. One-way ANOVA followed by Tukey’s honest significant difference (HSD) test with 95% confidence intervals for comparing more than three means were evaluated using IBM SPSS for Windows, Version 22.0 (IBM Corp., Armonk, NY, USA).

## 4. Conclusions

An eco-friendly, sunlight-induced green extracellular synthesis of AgNPs using the endophytic bacterium, *P. ananatis* has been reported in the present study. Extracellular extract of *P. ananatis* under exposure to sunlight increases the intensity of the surface plasmon resonance peak of the AgNPs and successfully stabilizes the silver nanoparticles in dispersion. FTIR spectra and TEM images confirm the role of the extract as the capping and stabilizing agents of the synthesized AgNPs, respectively. The hydrodynamic diameter of the synthesized AgNPs indicates a better control of their size and polydispersity. The synthesized AgNPs demonstrate effective antimicrobial efficacy against pathogenic microbes as well as MDR bacteria under the tested experimental conditions. The exploration and use of endophytic bacteria as a source of cheap biomaterial provides an opportunity to develop a facile, eco-friendly, cost-effective and rapid synthesis of stable and polydispersed AgNPs, which have the potential to be used as the promising antimicrobial agents against pathogenic as well as MDR microbes to combat the menace of antibiotic resistance. However, further studies are warranted to make the biosynthesized AgNPs as a safe therapeutic agent in the modern era of medicine.

## Figures and Tables

**Table 1:** Elemental composition of the synthesized AgNPs.

Element Number	Element Symbol	Element Name	Atomic Conc.	Weight Conc.
6	C	Carbon	70.08	49.02
47	Ag	Silver	3.74	23.50
8	O	Oxygen	23.46	21.86
17	Cl	Chlorine	2.72	5.61

**Table 2:** Zeta potential value of the synthesized AgNPs.

Triplicate Experiments with the Synthesized AgNPs	Zeta Potential (ζ) (mV)	Mean (mV)	Area (%)	Conductivity (mS/cm)
1	−6.75	3.49	100	0.0127
2	−7.92	3.54	100	0.0129
3	−7.77	2.97	100	0.0355

**Table 3:** ZOI (mm) and MIC (μg/mL) values of the synthesized AgNPs against pathogenic microbes and MDR bacteria.

**Pathogenic Microbes ^1^**		
**Microorganisms**	**Synthesized AgNPs**	**Control**
*B. cereus* (ATCC 10876)	9.16 ± 0.05 ^c^ (2.25)	Ampicilin (10 μg): resistant
*S. aureus* subsp. *aureus* (ATCC 11632)	11.30 ± 0.07 ^b^ (2.75)	Ampicilin (10 μg): 10.14±0.05
*E. coli* (ATCC 10536)	15.12 ± 0.08 ^a^ (3.25)	Ciprofloxacin (5 μg): 30.48±0.08
*P. aeruginosa* (ATCC 10145)	8.02 ± 0.08 ^d^ (1.75)	Ciprofloxacin (5 μg): 30.10±0.07
*C. albicans* (ATCC 10231)	7.16 ± 0.09 ^e^ (1.75)	Itraconazole (10 μg): resistant
**MDR bacteria ^2^**		
*S. pneumoniae* (ATCC700677)	10.20 ± 0.07 ^B^ (2.75)	-
*E. faecium* (ATCC 700221)	12.16 ± 0.05 ^A^ (2.25)	-
*S. aureus* subsp. *aureus* (ATCC33592)	10.16 ± 0.05 ^B^ (3.75)	-
*E. coli* (NCTC 13351)	12.24 ± 0.05 ^A^ (3.50)	-

^1^ AgNPs concentration: 300 μg/mL (6 μg/disc). ^2^ AgNPs concentration: 500 μg/mL (10 μg/disc). Results are observed as mean±SD. Significant differences among the mean values of the pathogenic microbes, and MDR bacteria determined by the Tukey′s HSD test (*p* < 0.05) are indicated by different letters (^a–e^ and ^A–B^, respectively).
